# Denial as an ethical problem: the example of ICU triage in the context of the COVID-19 pandemic

**DOI:** 10.1515/ajmedh-2024-0009

**Published:** 2025-01-07

**Authors:** Yanick Farmer, Marie-Eve Bouthillier

**Affiliations:** Department of Social and Public Communication, Université du Québec à Montréal, Montreal, Quebec, Canada; Faculté de Medicine, Université de Montréal, Montréal, Québec, Canada

**Keywords:** denial, clinical ethics, psychology, triage, COVID-19

## Abstract

**Objectives:**

The overall goal of this article is to show that denial is one of the greatest obstacles to good practical judgment and is therefore a major problem in clinical ethics by examining its cognitive structure and the challenges it poses for clinical ethics consultation and intervention. In addition to clinical examples, excerpts of verbatim from citizen forums on triage protocols will be used to illustrate the manifestations of denial in citizens when faced with difficult choices.

**Case presentation:**

The initial waves of the pandemic and the alarming resurgence of cases with the emergence of highly transmissible variants have created increased pressure on many healthcare systems around the world. These critical situations have activated the potential for health authorities in different countries to use triage protocols to manage access to critical care. In several cases, public opinion was alerted, creating a climate of concern and even suspicion among the general population. These debates have highlighted both the importance and the difficulty of basing triage choices and the allocation of scarce resources on an ethical or moral reasoning that commands strong support. The obstacles to this consensus are numerous. There is, of course, the diversity of beliefs and values, but also a mechanism that has been very little documented in clinical ethics: denial.

**Conclusions:**

Denial poses major problems for providers and professionals in healthcare settings. In the face of maladaptive behaviors such as denial, psychotherapy uses techniques that act on both the cognitive and affective levels. Many of these techniques require long-term work that can only be accomplished in the context of professionally supervised therapy, but some tips can be identified for mediation and the work of the clinical ethicist.

## Introduction

Among the many negative effects of the COVID-19 pandemic on countries around the world, the overflow of emergency departments in hospitals is probably one of the issues that has attracted the attention of governments and health authorities the most [1]. One of the consequences of the sudden massive influx of new patients to the emergency department due to severe acute respiratory syndrome was the increasing need for mechanical ventilators in intensive care units to assist with breathing for patients whose lungs were attacked by the virus [2]. Given the sudden increase in need and the limited capacity of hospitals to meet this need, several countries have taken the initiative to create triage protocols that aim to establish prioritization criteria for access to intensive care in extreme pandemic contexts [3].

Triage protocols are used to decide who will have priority access to resources. Established criteria most often include clinical prognosis and chances of survival, comorbidities, and frailties arising from patients’ overall health status [4]. In addition to clinical criteria, protocols also typically address the logistical challenges associated with their implementation such as the formation of prioritization teams and the redirection of non-ICU patients to other care units. Several protocols also include criteria for deciding on clinical ties between patients (tie breakers) [5], 6].

The initial waves of the pandemic and the alarming resurgence of cases during the emergence of highly transmissible variants such as Delta created increased pressure on many healthcare systems around the world [7]. These critical situations have activated the potential for health authorities in different countries to use triage protocols to manage access to critical care. In several cases, public opinion was alerted, creating a climate of concern and even suspicion among the general population. Among the most recurrent negative comments was the idea that triage protocols would potentially discriminate against certain categories of patients such as the elderly or those with disabilities. According to these critics, these individuals would no longer have equal access to care [8], 9].

These debates have highlighted both the importance and the difficulty of basing choices about triage and the allocation of scarce resources on ethical or moral reasoning that commands strong support. The barriers to achieving broad social consensus on the ethical principles, which should be applied to triage in critical care, have a variety of causes. For example, the pluralism of beliefs and values resulting from Hume’s is-ought problem states that it is not possible to derive a normative prescription from a judgment of fact [10], which may account for much of the challenge of reaching consensus. When triage protocols were debated in the media, much of the disagreement stemmed from how to integrate clinical (factual; what is) data into moral reasoning (what ought to be). In philosophy and meta-ethics, these questions and the numerous reflections they provoke are quite well known.

However, our recent empirical work in citizen forums on prioritization protocols and their underlying ethical values [11] indicates that an extremely significant, yet insufficiently documented phenomenon in clinical ethics is also at work: *denial.* Denial is a defense mechanism that acts in response to inner conflicts and stress. It can be defined as a refusal to acknowledge certain painful aspects of external reality or subjective experience that would be obvious to others [12]. In our work and in several clinical ethics consultations we have conducted over the years in health care settings, it has become apparent to us that denial is the source of many interpretive conflicts that prevent groups of individuals from agreeing on the best decisions to make in the face of a given problem [13]. *The thesis we wish to defend in this article is that denial is one of the greatest obstacles to good practical judgment and is therefore a major problem in clinical ethics.*

Two aspects of the phenomenon will be examined in the remainder of this article to support this claim. The first concerns the cognitive mechanisms of denial and the ways in which denial obscures practical judgment. The second relates to the difficulty of removing denial in the context of clinical ethics consultation. We will conclude our reflection with a broader discussion of avenues to explore for improving professional practice in the face of denial.

## The psychology of denial

As a defense mechanism, denial aims to eliminate the source of discomfort by diverting the perception of certain information. As the scientific literature in psychology abundantly demonstrates, the human brain very rarely follows a purely rational logic when reasoning, processing information, and making decisions. There are a host of phenomena, such as bias and cognitive dissonance, that account for the fact that emotions and personal beliefs play a powerful role in cognitive and behavioral processes. For example, this is the case in one of the best-known forms of bias: *confirmation bias*. The APA dictionary defines confirmation bias as “a cognitive bias that favors information that confirms your previously existing beliefs” [14]. In this phenomenon, information processing is strongly constrained by beliefs that the individual seeks to maintain. This situation is homologous to what we find in *cognitive dissonance*. This manifests itself as “an unpleasant psychological state resulting from inconsistency between two or more elements in a cognitive system” [15]. The psychologist Leon Festinger was the first to describe this phenomenon, and stated that this discomfort can take the form of a contradiction between a perceived fact and a deeply held belief [16]. The discomfort then leads the individual to reduce the dissonance through various means such as justification or rationalization of objectively inadequate behaviors.

In addition to psychology, other fields have taken an interest in denial. For example, from an anthropological and sociological perspective, Ernest Becker explains in *The Denial of Death* [17] that the innate *fear of death* has driven human beings to create powerful cultural and symbolic systems aimed at transcending the inescapable end of life. The mythologies surrounding heroism and the promises of immortality made by the great religions are both ancient and contemporary expressions of an original denial of the finitude and limits of existence. Becker goes on to assert that the hedonistic culture focused on the pursuit of carnal pleasures is a way of fleeing from death. This conception of denial is certainly noteworthy, but it primarily invites us to understand this phenomenon from the collective perspective of societies. The perspective we aim to develop in this article focuses on the clinical manifestation in the context of healthcare delivery. From this viewpoint, psychology offers a finer understanding of the cognitive mechanisms that accompany denial, which in turn facilitates the creation of intervention tools in clinical ethics.

The scientific literature in psychology associates denial with the cognitive mechanism known as *motivated reasoning*. This notion implies that the human brain often bases its inferences not on evidence, but rather on personal goals. This phenomenon goes beyond *wishful thinking* [18]. It prompts the individual not only to believe something that is not based on a full analysis of the facts, but also to try to find evidence and arguments that support goals or motivations that are often unconscious. For example, a person who wants to be in a romantic relationship to fill a gap in affection might justify the value of their partner despite signals that they should be wary. Another person might seek to minimize the impact of using their polluting vehicle by claiming that climate change is not really man-made [19].

In our research with citizens who spoke out about which values to prioritize in triage protocols, our research team was struck by the extent to which denial prevents some individuals from making choices that may seem obvious. Some even tried to avoid or circumvent the questions they were asked. For example, the criterion of clinical prognosis and efficient use of critical care resources was challenged by people who were sensitive to the risks of discrimination against certain categories of patients. Nevertheless, there is scientific evidence that very frail patients with advanced disease may not benefit from invasive mechanical ventilation due to higher mortality and longer ICU stay [20].

Denial was not part of our research questions, but this theme emerged in discussions with citizens. Participants were randomly solicited by a polling firm from a pool of 370 people. Two groups of 30 citizens were then selected to participate in two separate 2-day forums, one in English and one in French. Criteria of representativeness (age, gender, origin, education, etc.) were applied in the constitution of these two groups. Day 1 (May) of the forum consisted of an information session given by experts on triage protocols and the issues involved. Day 2 (June) was to get the public to discuss the acceptability of triage protocols and to identify the conditions and considerations they should incorporate to be ethically justifiable from the public’s point of view. On Day 1, the public didn’t seem to realize that we wanted them to discuss the case where there was a real shortage of intensive care places and tragic choices had to be made. They avoided the issue by continually proposing “solutions” based on increasing resources when we were saying that there were none left. On Day 2, we had no choice but to reframe the discussion using a disaster scenario (clinical vignette) to tell a story as if someone were arriving at the hospital in a context where there was no more beds. It was only then that something clicked and they collectively came out of the denial of the disaster scenario. The following qualitative analysis of denial expressions from citizens participating at the forums is not systematic. We just present a few excerpts to illustrate how denial manifests itself in relation to the issue of triage.

Table 1 shows some quotes from what citizens said (verbatim) at the triage protocol forums. Some of them have been freely translated from French.

**Table 1:** Examples of denial expressions from citizens participating at the forums.

Extracts from verbatim (citizens forums, May and June 2022)
*“My opinion is that we should not choose. We must do everything to save everyone without distinction.”*
*“Again, everyone should have the chance to survive. We have to fight for them.”*
*“Too hard to choose. I want to save them both. Can we set up an extra bed? And the two of them can share the machine maybe?”*
*“In my opinion, you are not proposing the right options. We need to find solutions to save everyone, not choose who should live or die.”*
After deliberations, the majority of citizens supported the idea of a prioritization protocol based on clinical prognosis, but some remained adamant:
*“I am still with the same opinion that we must try to find other solutions instead of considering the one that says that we must sacrifice some to save other lives. I believe that we must find a way to improve our health system (more doctors, nurses or others) and also have more medical machines and materials. I believe that all the financial means should be invested on the purchase of all that can improve the number of lives that we can save.”*

Among the most recognized and controversial sources of conflict in critical care are the demands of loved ones to administer life-sustaining care, even when clinicians believe these interventions are futile [21], 22]. Such situations illustrate the understandable but irrational desire of individuals to do whatever they can to provide their loved ones with the same life-sustaining resources as others, even if it is unrealistic. The argument they develop follows the structure of denial as we have defined it. First, they underestimate the obstacles to intervention represented by the clinical condition of their loved ones (incorrect inference based on biased information processing). Secondly, they support their reasoning with an ethical argument based on the pre-eminence of values such as respect for equality and dignity. However, the imposition of inappropriate treatment can lead to therapeutic obstinacy, and thus to a form of violation of the patient’s dignity, while undermining the principle of responsible use of resources, which is vital in the extreme context of a pandemic.

## The challenges of intervention and clinical ethics consultation

Denial poses major problems for providers and professionals in healthcare settings. Effective health communication normally requires healthcare professionals to be able to convey information clearly and respectfully of the patient’s literacy [23], 24]. However, it becomes difficult to communicate adequately without the presence of common referents. Indeed, for communication to be effective, it is important that the way in which certain facts are interpreted and referred to is clear [25], 26]. This is not always possible when a person is in denial. As we have seen, denial results in a significant distortion in the processing of information due to more or less conscious emotions, beliefs, or goals. Since the principles of effective communication focus primarily on the transmission of information, upstream issues related to the emotional underpinnings of denial often remain unresolved [27].

There are many examples of typical cases of denial in healthcare settings. For example, in our clinical ethics practice, we have seen a family in which several children were physicians. They harass their father’s attending physician to continue intubating him when his advanced and irreversible medical condition had reduced him to a living skeleton. Still, others refuse to acknowledge their medical condition, even to a very advanced stage of cancer, pregnancy, or other. Given the life-and-death stakes involved in accessing intensive care in the event of complications from COVID 19, it is understandable that denial is common when addressing the issue of triage.

## Discussion

In general, the sparse scholarly literature devoted to the issue of denial and health ethics is limited to noting its harmful effects on the care relationship [28], [29], [30]. Very few focus on methods of thinking about the practice of clinical ethics consultation, from a description of denial and its underlying psychological mechanisms [31], [32], [33], [34] Our investigation of the denial that is expressed in the general population in the face of the triage process invites us to go beyond these barriers and also imagine how clinical ethics consultation can touch on unconscious motivations that distort perceptions of reality.

There are different streams of clinical psychology that focus on denial and cognitive biases. Among these, cognitive-behavioral therapies are based precisely on the idea that many psychological problems result from faulty information processing and maladaptive behaviors [35]. Within this family of therapies, some approaches such as cognitive bias modification of interpretation (CBM-I) aim to “undo” the harmful effects of bias [36]. While it is not the role of the clinical ethicist to turn into a psychologist or a psychiatrist, the fact remains that he or she can find useful insights for practice in psychological research [27].

In the face of maladaptive behaviors such as denial, psychotherapy uses techniques that act on both the cognitive and affective levels. Many of these techniques require long-term work that can only be accomplished in the context of professionally supervised therapy, but some tips can be identified for mediation and the work of the clinical ethicist. For example, to reinforce the desire to change the misperceptions associated with denial, it is first necessary to create awareness [37]. To accomplish this, it is important to demonstrate empathy and compassion to create a climate that is conducive to mediation, an approach that can be used in difficult clinical ethics consultations [37], 38]. A respectful attitude and appropriate body language help to make this empathy visible.

When this climate of trust is established, it becomes easier to move the person – who may be accompanied by those around them – towards an exploration of the facts and arguments that contradict the belief, motivation, or goal that impedes an adequate perception of the facts. In this process, it may become necessary to reframe the discussion to prevent avoidance of what may be felt as potentially painful. One of the techniques proposed by psychotherapy to facilitate the work of awareness and reframing relates to the cost-benefit analysis of maladaptive thought patterns (beliefs, motivations, etc.). “This technique is intended to increase motivation for change by exploring the present and past benefits, but also the negative impacts of a given pattern” [37]. In a clinical ethics consultation, this may involve meeting the person and their loved ones and exploring, through open questions, what they understand about the situation and putting at the heart of the discussion what is most important to them. Giving them the space to tell their story and, through empathic feedback, helping the story to evolve towards a shared understanding. Without coercion, this exercise can help people to become aware of the consequences of certain decisions, and to revise certain points of view if necessary.

Techniques that only act on the cognitive aspect of denial may not be sufficient because motivation draws its resources from a deeper emotional level. In the context of simple clinical ethics consultation, it is not primarily the role of the clinical ethicist to act decisively at this level. However, a “narrative and humanistic” approach to intervention [39] and clinical ethics consultation [40] allows for the expression of emotions from a perspective of acceptance and empathy, which can help identify the “triggers” of this defense mechanism that is denial and move towards awareness and change [37]. The humanistic approach attaches particular importance to the bond created between the practitioner and the person through respect, active (reflective) listening and empathy.

Our research shows that when asked about triage, some citizens reveal reasons for pain (e.g., having a loved one who faces the daily challenges of a disability) that may explain the denial. If our work has been able to highlight the extent to which denial is a major issue in clinical ethics, further investigation is certainly needed to organize these findings and integrate them into clinical ethics consultation practices.

## References

[1] Badalov E, Blackler L, Scharf AE, Matsoukas K, Chawla S, Voigt LP (2022). COVID-19 double jeopardy: the overwhelming impact of the social determinants of health. Int J Equity Health.

[2] Wunsch H (2020). Mechanical ventilation in COVID-19: interpreting the current epidemiology. Am J Respir Crit Care Med.

[3] White DB, Lo B (2021). Mitigating inequities and saving lives with ICU triage during the COVID-19 pandemic. Am J Respir Crit Care Med.

[4] Tyrrell CSB, Mytton OT, Gentry SV, Thomas-Meyer M, Allen JLY, Narula AA (2021). Managing intensive care admissions when there are not enough beds during the COVID-19 pandemic: a systematic review. Thorax.

[5] Aquino YSJ, Rogers WA, Scully JL, Magrabi F, Carter SM (2022). Ethical guidance for hard decisions: a critical review of early international COVID-19 ICU triage guidelines. Health Care Anal.

[6] Piscitello GM, Kapania EM, Miller WD, Rojas JC, Siegler M, Parker WF (2020). Variation in ventilator allocation guidelines by US state during the coronavirus disease 2019 pandemic: a systematic review. JAMA Netw Open.

[7] Dol J, Boulos L, Somerville M (2022). Health system impacts of SARS-CoV-2 variants of concern: a rapid review. BMC Health Serv Res.

[8] Felt AB, Mitcham D, Hathcock M, Swienton R, Harris C (2022). Discrimination and bias in state triage protocols toward populations with intellectual disabilities during the COVID-19 pandemic. Disaster Med Public Health Prep.

[9] Riva L, Petrini C (2021). Ethics of triage for intensive-care interventions during the COVID-19 pandemic: age or disability related cut-off policies are not justifiable. Clin Ethics.

[10] Hume D, Coventry A (2023). A treatise of human nature: being an attempt to introduce the experimental method of reasoning into moral subjects.

[11] Ramirez CC, Farmer Y, Bouthillier ME (2023). Public voices on tie-breaking criteria and underlying values in COVID-19 triage protocols to access critical care: a scoping review. Discov Health Syst.

[12] American Psychological Association (2023). Denial. ..

[13] Blumenthal-Barby JS, Ubel PA (2018). In defense of “denial”: difficulty knowing when beliefs are unrealistic and whether unrealistic beliefs are bad. Am J Bioeth.

[14] Cherry K (2022). What is the confirmation bias?.

[15] Cherry K (2022). What is cognitive dissonance?.

[16] Festinger L (1957). A theory of cognitive dissonance.

[17] Becker E (1973). The denial of death.

[18] Bastardi A, Uhlmann EL, Ross L (2011). Wishful thinking: belief, desire, and the motivated evaluation of scientific evidence: belief, desire, and the motivated evaluation of scientific evidence. Psychol Sci.

[19] Thagard P (2021). The cognitive science of COVID-19: acceptance, denial, and belief change. Harv Public Health Rev.

[20] Okahara S, Subramaniam A, Darvall JN, Ueno R, Bailey M, Pilcher DV (2022). The relationship between frailty and mechanical ventilation: a population-based cohort study. Ann Am Thorac Soc.

[21] Frechman E, Dietrich MS, Walden RL, Maxwell CA (2020). Exploring the uptake of advance care planning in older adults: an integrative review. J Pain Symptom Manag.

[22] Bosslet GT, Pope TM, Rubenfeld GD, Lo B, Truog RD, Rushton CH (2015). An official ATS/AACN/ACCP/ESICM/SCCM policy statement: responding to requests for potentially inappropriate treatments in intensive care units. Am J Respir Crit Care Med.

[23] Ratna H (2019). The importance of effective communication in healthcare practice. Harv Publ Health Rev.

[24] Ha JF, Longnecker N (2010). Doctor-patient communication: a review. Ochsner J.

[25] Allan K (2013). What is common ground?. Perspectives in pragmatics, philosophy & psychology.

[26] Clark HH, Brown K (2006). Context and common ground. Concise encyclopedia of philosophy of language and linguistics.

[27] Childers JW, Arnold RM (2018). “I know I’m going to beat this”: when patients and doctors disagree about prognosis. Am J Bioeth.

[28] Aggarwal N, Rowe M (2013). Denial in patient-physician communication among patients with cancer. New challenges in communication with cancer patients.

[29] Jenkins A, Millar S, Robins J (2011). Denial of pregnancy-a literature review and discussion of ethical and legal issues. J Roy Soc Med.

[30] Rabinowitz T, Peirson R (2006). “Nothing is wrong, doctor”: understanding and managing denial in patients with cancer. Cancer Invest.

[31] Guerin RM (2022). Mechanisms of defense in clinical ethics consultations. Med Healthc Philos.

[32] Magelssen M, Pedersen R, Førde R (2014). Sources of bias in clinical ethics case deliberation. J Med Ethics.

[33] Agich GJ (2011). Defense mechanisms in ethics consultation. HEC Forum.

[34] Schleger A, Oehninger H, Reiter-Thell NR (2011). Avoiding bias in medical ethical decision-making. Lessons to be learnt from psychology research. Med Health Care Philos.

[35] American Psychological Association (2017). What is cognitive behavioural therapy?.

[36] Krebs G, Pile V, Grant S, Degli Esposti M, Montgomery P, Lau JYF (2018). Research review: cognitive bias modification of interpretations in youth and its effect on anxiety: a meta-analysis. J Child Psychol Psychiatry.

[37] Gauthier C, Chaloult G, Goulet J (2019). Guide pratique pour la théorie des schémas.

[38] Fiester A (2015). Contentious conversations: using mediation techniques in difficult clinical ethics consultations. J Clin Ethics.

[39] Rogers CR (1974). A theory of therapy and personality change: as developed in the client-centered framework. Perspectives in abnormal behavior.

[40] Agich GJ (2018). Narrative and method in ethics consultation. Peer review, peer education, and modeling in the practice of clinical ethics consultation: the Zadeh project.

